# Tetra­ethyl­ammonium tri-μ-phenolato-bis­[tricarbonyl­manganate(I)]

**DOI:** 10.1107/S1600536811029266

**Published:** 2011-07-30

**Authors:** Timothy J. McNeese, Robert D. Pike

**Affiliations:** a Department of Chemistry, Loyola University Maryland, 4501 N. Charles Street, Baltimore, MD 21210-2699, USA; b Department of Chemistry, College of William and Mary, PO Box 8795, Williamsburg, VA 23187-8795, USA

## Abstract

The title compound, (C_8_H_20_N)[Mn_2_(C_6_H_5_O)_3_(CO)_6_], was synthesized from [Mn(CO)_3_(CH_3_CN)_3_]BF_4_ and (C_8_H_20_N)(OC_6_H_5_). The binuclear anion exhibits a pseudo-threefold symmetry and contains two six-coordinate Mn atoms. Each metal atom is coordinated by three facially oriented CO ligands and three doubly-bridging phenolate ligands. The average O—Mn—O bond angle is 74.9 (7)° in the Mn_2_O_3_ metal–phenolate dimeric core, yielding a distorted octa­hedron for each metal.

## Related literature

For the synthesis of the starting materials, see: Riemann & Singleton (1973 ▶); McNeese *et al.* (1985 ▶). For related metal phenolate complexes, see: Darensbourg *et al.* (1988 ▶, 1989 ▶); McNeese *et al.* (1985 ▶); Lee *et al.* (1995 ▶). For analogous tungsten and rhenium dimers, see: Darensbourg *et al.* (1988 ▶); Beringhelli *et al.* (1985 ▶).
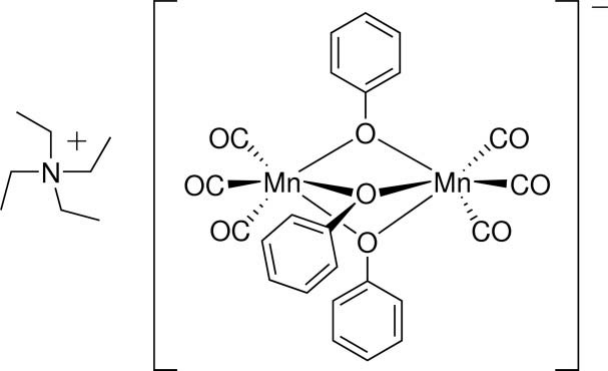


## Experimental

### 

#### Crystal data

(C_8_H_20_N)[Mn_2_(C_6_H_5_O)_3_(CO)_6_]
*M* *_r_* = 687.49
Orthorhombic, 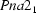

*a* = 18.6831 (4) Å
*b* = 9.2037 (2) Å
*c* = 18.5999 (4) Å
*V* = 3198.32 (12) Å^3^
*Z* = 4
Cu *K*α radiation
μ = 6.88 mm^−1^
*T* = 100 K
0.45 × 0.43 × 0.30 mm

#### Data collection

Bruker SMART APEXII CCD diffractometer
Absorption correction: numerical (*SADABS*; Sheldrick, 2004 ▶) *T*_min_ = 0.148, *T*_max_ = 0.229
30613 measured reflections
4817 independent reflections
4660 reflections with *I* > 2σ(*I*)
*R*_int_ = 0.059

#### Refinement

*R*[*F* ^2^ > 2σ(*F* ^2^)] = 0.033
*wR*(*F* ^2^) = 0.084
*S* = 1.07
4817 reflections
402 parameters
19 restraints
H-atom parameters constrained
Δρ_max_ = 0.79 e Å^−3^
Δρ_min_ = −0.57 e Å^−3^
Absolute structure: Flack (1983 ▶), 1874 Friedel pairs
Flack parameter: 0.051 (5)

### 

Data collection: *APEX2* (Bruker, 2004 ▶); cell refinement: *SAINT-Plus* (Bruker, 2004 ▶); data reduction: *SAINT-Plus*; program(s) used to solve structure: *SHELXS97* (Sheldrick, 2008 ▶); program(s) used to refine structure: *SHELXL97* (Sheldrick, 2008 ▶); molecular graphics: *ORTEP-3* (Farrugia, 1997 ▶) and *Mercury* (Macrae *et al.*, 2006 ▶); software used to prepare material for publication: *SHELXL97*.

## Supplementary Material

Crystal structure: contains datablock(s) I, global. DOI: 10.1107/S1600536811029266/bg2415sup1.cif

Structure factors: contains datablock(s) I. DOI: 10.1107/S1600536811029266/bg2415Isup2.hkl

Additional supplementary materials:  crystallographic information; 3D view; checkCIF report

## Figures and Tables

**Fig. 1.**
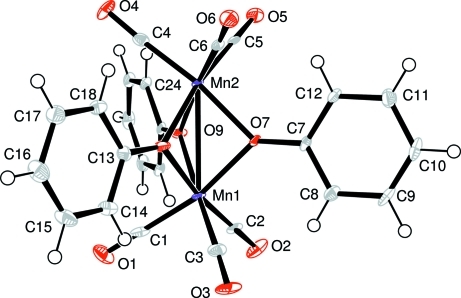
Ellipsoid plot of the organometallic anion in (1). Displacement ellipsoids are drawn at the 50% probability level.

**Fig. 2.**
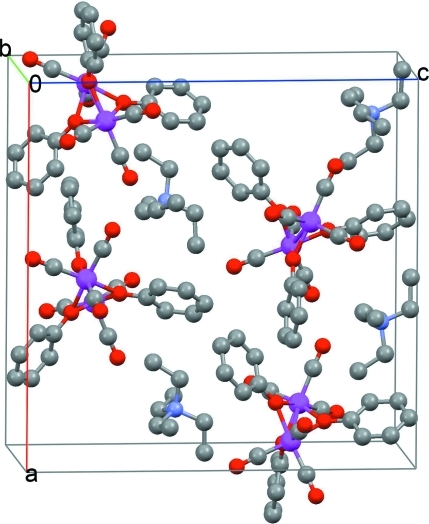
Packing diagram of (1) viewed down the b axis.

## supplementary crystallographic information

### Comment

The phenolate ligand, OC_6_H_5_^-^, exhibits a variety of bonding modes in organometallic carbonyl compounds. Examples include mono- and polynuclear complexes where the ligand is bonded by its oxygen atom to one or more metals in a terminal (Darensbourg *et al.*, 1989), doubly- (Darensbourg *et al.*, 1988) and triply- (McNeese, *et al.*, 1985) bridging fashion, and through its aromatic ring as an oxocyclohexadienyl ligand (Lee *et al.*, 1995).

The title compound, (Et_4_N)[(CO)_3_Mn(µ-OC_6_H_5_)_3_Mn(CO)_3_] (1), was synthesized by displacement of labile acetonitrile ligands of the precursor, [Mn(CO)_3_(CH_3_CN)_3_]BF_4_ (Riemann & Singleton, 1973), by phenoxide ions of (Et_4_N)(OC_6_H_5_) (McNeese *et al.*, 1985). The doubly-bridging phenolate ligands of the dimer are basic, reaction of (1) in CH_3_CN with HBF_4_ re-forms the Mn-containing starting material and C_6_H_5_OH.

The bimetallic compound crystallizes in the orthorhombic space group Pna2(1), and exhibits a striking pseudo threefold symmetry. The structure presents bond lengths and angles that are comparable to analogous Re (Beringhelli *et al.*, 1985) and W (Darensbourg *et al.*, 1988) dimers. The geometry for each Mn atom in the organometallic anion of (1) is a distorted octahedron (Figure 1), with average O—Mn—O angles in the Mn_2_O_3_ metal-phenolate group of 75.12 (7)°. Each Mn atom has a noble-gas configuration and a nonbonding metal distance of 2.8759 (5) Å. Figure 2 illustrates the packing diagram for the molecular structure of the title compound.

### Experimental

Solid (Et_4_N)(OC_6_H_5_) (500 mg, 2.24 mmol) (McNeese *et al.*, 1985) was added with a funnel to a 100-ml Schlenk tube containing a 50-ml CH_3_CN solution of [Mn(CO)_3_(CH_3_CN)_3_]BF_4_ (521 mg, 1.49 mmol) (Riemann & Singleton, 1973). The orange-colored solution was stirred under argon at room temperature for 18 h and evaporated to dryness. THF (25 ml) was added to the solid to dissolve the desired compound. The reaction mixture was filtered under vacuum to separate insoluble Et_4_NBF_4_ and the resulting solution was evaporated. The orange-colored product, (Et_4_N)[(CO)_3_Mn(µ-OC_6_H_5_)_3_Mn(CO)_3_], was crystallized from THF/cyclohexane. Yield: 359 mg (69%). IR (υ(CO), CH_3_CN) 2013 (*s*), 1912 (*s*) cm^-1^; ^1^H NMR (CD_3_CN): cation, δ1.25 (12*H*,t, –CH_3_), 3.25 (8*H*, q, –CH_2_–); anion, δ 6.75–7.45 (15*H*, m, OC_6_H_5_). Elemental analysis, calcd for C_32_H_35_Mn_2_NO_9_: C, 55.90; H, 5.14. Found: C, 55.67; H, 5.28.

### Refinement

All hydrogen atoms were placed in theoretical positions (C—H: 0.95–0.99 Å) riding on the atoms to which they are attached.

Displacement factors of the atoms attached to Mn were restarined *via* DELU intructions in *SHELXL*.

### Crystal data

**Table:**

(C_8_H_20_N)[Mn_2_(C_6_H_5_O)_3_(CO)_6_]	*F*(000) = 1424
*M**_r_* = 687.49	*D*_x_ = 1.428 Mg m^−^^3^
Orthorhombic, *P**n**a*2_1_	Cu *K*α radiation, λ = 1.54178 Å
Hall symbol: P 2c -2n	Cell parameters from 9989 reflections
*a* = 18.6831 (4) Å	θ = 3.4–68.1°
*b* = 9.2037 (2) Å	µ = 6.88 mm^−^^1^
*c* = 18.5999 (4) Å	*T* = 100 K
*V* = 3198.32 (12) Å^3^	Block, brown
*Z* = 4	0.45 × 0.43 × 0.30 mm

### Data collection

**Table:**

Bruker SMART APEXII CCD diffractometer	4817 independent reflections
Radiation source: fine-focus sealed tube	4660 reflections with *I* > 2σ(*I*)
graphite	*R*_int_ = 0.059
ω and ψ scans	θ_max_ = 67.0°, θ_min_ = 4.7°
Absorption correction: numerical (*SADABS*; Sheldrick, 2004)	*h* = −22→22
*T*_min_ = 0.148, *T*_max_ = 0.229	*k* = −10→10
30613 measured reflections	*l* = −19→22

### Refinement

**Table:**

Refinement on *F*^2^	Secondary atom site location: difference Fourier map
Least-squares matrix: full	Hydrogen site location: inferred from neighbouring sites
*R*[*F*^2^ > 2σ(*F*^2^)] = 0.033	H-atom parameters constrained
*wR*(*F*^2^) = 0.084	*w* = 1/[σ^2^(*F*_o_^2^) + (0.0591*P*)^2^] where *P* = (*F*_o_^2^ + 2*F*_c_^2^)/3
*S* = 1.07	(Δ/σ)_max_ = 0.001
4817 reflections	Δρ_max_ = 0.79 e Å^−^^3^
402 parameters	Δρ_min_ = −0.57 e Å^−^^3^
19 restraints	Absolute structure: Flack (1983), 1874 Friedel pairs
Primary atom site location: structure-invariant direct methods	Flack parameter: 0.051 (5)

### Special details

**Table:**

Geometry. All e.s.d.'s (except the e.s.d. in the dihedral angle between two l.s. planes) are estimated using the full covariance matrix. The cell e.s.d.'s are taken into account individually in the estimation of e.s.d.'s in distances, angles and torsion angles; correlations between e.s.d.'s in cell parameters are only used when they are defined by crystal symmetry. An approximate (isotropic) treatment of cell e.s.d.'s is used for estimating e.s.d.'s involving l.s. planes.
Refinement. Refinement of *F*^2^ against ALL reflections. The weighted *R*-factor *wR* and goodness of fit *S* are based on *F*^2^, conventional *R*-factors *R* are based on *F*, with *F* set to zero for negative *F*^2^. The threshold expression of *F*^2^ > σ(*F*^2^) is used only for calculating *R*-factors(gt) *etc*. and is not relevant to the choice of reflections for refinement. *R*-factors based on *F*^2^ are statistically about twice as large as those based on *F*, and *R*- factors based on ALL data will be even larger.

### Fractional atomic coordinates and isotropic or equivalent isotropic displacement parameters (Å^2^)

**Table:**

	*x*	*y*	*z*	*U*_iso_*/*U*_eq_	
Mn1	0.942524 (18)	0.23445 (4)	0.69042 (3)	0.00987 (11)	
Mn2	0.864233 (19)	0.49426 (4)	0.72511 (3)	0.00854 (11)	
O1	0.88111 (12)	−0.0575 (2)	0.71027 (13)	0.0277 (5)	
O2	1.00084 (11)	0.1572 (2)	0.54831 (12)	0.0255 (5)	
O3	1.07657 (10)	0.1197 (2)	0.75028 (13)	0.0253 (5)	
O4	0.71971 (11)	0.5205 (2)	0.78704 (13)	0.0246 (5)	
O5	0.82718 (11)	0.7131 (2)	0.61509 (12)	0.0197 (4)	
O6	0.90503 (11)	0.7115 (2)	0.83376 (12)	0.0194 (4)	
O7	0.96310 (8)	0.45028 (19)	0.68333 (11)	0.0120 (4)	
O8	0.89854 (9)	0.3187 (2)	0.78004 (11)	0.0139 (4)	
O9	0.85015 (9)	0.32527 (19)	0.65656 (11)	0.0118 (4)	
C1	0.90466 (14)	0.0552 (3)	0.70182 (17)	0.0151 (5)	
C2	0.97751 (12)	0.1871 (3)	0.60351 (16)	0.0134 (5)	
C3	1.02446 (13)	0.1687 (3)	0.72812 (18)	0.0160 (5)	
C4	0.77549 (14)	0.5058 (3)	0.76187 (18)	0.0133 (5)	
C5	0.84219 (13)	0.6313 (3)	0.65873 (15)	0.0114 (5)	
C6	0.89061 (13)	0.6281 (3)	0.79007 (15)	0.0110 (5)	
C7	1.02389 (13)	0.5276 (3)	0.67916 (14)	0.0101 (5)	
C8	1.08961 (14)	0.4598 (3)	0.66590 (16)	0.0156 (6)	
H8	1.0915	0.3575	0.6593	0.019*	
C9	1.15232 (15)	0.5415 (4)	0.66233 (17)	0.0191 (6)	
H9	1.1965	0.4939	0.6531	0.023*	
C10	1.15143 (15)	0.6886 (3)	0.67177 (17)	0.0212 (6)	
H10	1.1947	0.7428	0.6708	0.025*	
C11	1.08608 (16)	0.7580 (3)	0.68280 (19)	0.0196 (6)	
H11	1.0847	0.8606	0.6881	0.024*	
C12	1.02274 (13)	0.6785 (3)	0.68610 (16)	0.0141 (5)	
H12	0.9785	0.7273	0.6931	0.017*	
C13	0.89238 (14)	0.2703 (3)	0.84769 (16)	0.0134 (6)	
C14	0.92658 (15)	0.1421 (3)	0.86978 (17)	0.0186 (6)	
H14	0.9543	0.0884	0.8361	0.022*	
C15	0.92062 (16)	0.0925 (3)	0.93968 (18)	0.0232 (6)	
H15	0.9441	0.0052	0.9532	0.028*	
C16	0.88101 (18)	0.1679 (3)	0.99032 (18)	0.0244 (7)	
H16	0.8772	0.1331	1.0382	0.029*	
C17	0.84700 (17)	0.2953 (3)	0.96968 (17)	0.0209 (6)	
H17	0.8199	0.3487	1.0039	0.025*	
C18	0.85229 (14)	0.3455 (3)	0.89947 (16)	0.0156 (6)	
H18	0.8283	0.4325	0.8862	0.019*	
C19	0.79925 (13)	0.2836 (3)	0.60953 (16)	0.0104 (5)	
C20	0.81015 (12)	0.1642 (3)	0.56366 (16)	0.0137 (6)	
H20	0.8535	0.1106	0.5666	0.016*	
C21	0.75808 (14)	0.1237 (3)	0.51403 (16)	0.0176 (6)	
H21	0.7666	0.0431	0.4833	0.021*	
C22	0.69416 (14)	0.1987 (4)	0.50854 (18)	0.0193 (6)	
H22	0.6588	0.1708	0.4745	0.023*	
C23	0.68308 (13)	0.3167 (3)	0.55445 (17)	0.0175 (6)	
H23	0.6393	0.3689	0.5518	0.021*	
C24	0.73455 (14)	0.3590 (3)	0.60368 (16)	0.0143 (5)	
H24	0.7258	0.4404	0.6339	0.017*	
N1	0.62259 (13)	0.0037 (2)	0.90791 (16)	0.0168 (5)	
C25	0.70244 (15)	−0.0258 (4)	0.91741 (19)	0.0243 (7)	
H25A	0.7224	0.0490	0.9500	0.029*	
H25B	0.7082	−0.1212	0.9413	0.029*	
C26	0.74587 (17)	−0.0265 (4)	0.8490 (3)	0.0383 (9)	
H26A	0.7438	0.0698	0.8265	0.057*	
H26B	0.7263	−0.0990	0.8158	0.057*	
H26C	0.7957	−0.0506	0.8601	0.057*	
C27	0.58925 (14)	−0.1050 (3)	0.85663 (16)	0.0168 (6)	
H27A	0.5380	−0.0803	0.8506	0.020*	
H27B	0.6125	−0.0945	0.8091	0.020*	
C28	0.59471 (18)	−0.2619 (3)	0.87963 (19)	0.0251 (7)	
H28A	0.5685	−0.2757	0.9247	0.038*	
H28B	0.6451	−0.2875	0.8868	0.038*	
H28C	0.5741	−0.3243	0.8423	0.038*	
C29	0.58983 (18)	−0.0064 (3)	0.98216 (18)	0.0214 (7)	
H29A	0.6006	−0.1036	1.0023	0.026*	
H29B	0.6131	0.0666	1.0135	0.026*	
C30	0.5099 (2)	0.0171 (4)	0.9847 (2)	0.0307 (8)	
H30A	0.4862	−0.0537	0.9534	0.046*	
H30B	0.4988	0.1157	0.9681	0.046*	
H30C	0.4929	0.0048	1.0341	0.046*	
C31	0.61045 (16)	0.1516 (3)	0.87507 (17)	0.0205 (6)	
H31A	0.5585	0.1640	0.8669	0.025*	
H31B	0.6342	0.1539	0.8275	0.025*	
C32	0.6371 (2)	0.2799 (4)	0.9187 (2)	0.0340 (9)	
H32A	0.6888	0.2707	0.9262	0.051*	
H32B	0.6127	0.2817	0.9653	0.051*	
H32C	0.6271	0.3702	0.8927	0.051*	

### Atomic displacement parameters (Å^2^)

**Table:**

	*U*^11^	*U*^22^	*U*^33^	*U*^12^	*U*^13^	*U*^23^
Mn1	0.00445 (18)	0.0091 (2)	0.0160 (2)	0.00351 (13)	−0.00178 (18)	−0.00336 (17)
Mn2	0.00319 (18)	0.0078 (2)	0.0146 (2)	0.00217 (13)	0.00009 (17)	−0.00081 (15)
O1	0.0346 (11)	0.0128 (8)	0.0358 (15)	−0.0038 (8)	−0.0064 (10)	0.0000 (9)
O2	0.0132 (9)	0.0400 (13)	0.0232 (9)	0.0042 (9)	0.0009 (8)	−0.0140 (9)
O3	0.0156 (8)	0.0239 (11)	0.0364 (13)	0.0116 (7)	−0.0130 (9)	−0.0079 (9)
O4	0.0079 (8)	0.0358 (13)	0.0300 (14)	0.0031 (8)	0.0050 (8)	−0.0003 (9)
O5	0.0188 (10)	0.0174 (10)	0.0230 (11)	0.0053 (8)	0.0002 (9)	0.0044 (7)
O6	0.0192 (9)	0.0179 (10)	0.0211 (11)	−0.0012 (8)	0.0008 (9)	−0.0062 (7)
O7	0.0040 (7)	0.0110 (9)	0.0209 (11)	0.0000 (6)	0.0017 (8)	−0.0027 (8)
O8	0.0139 (8)	0.0117 (9)	0.0160 (10)	0.0058 (7)	0.0018 (8)	−0.0004 (7)
O9	0.0052 (8)	0.0104 (9)	0.0198 (11)	0.0020 (7)	−0.0046 (7)	−0.0042 (7)
C1	0.0124 (12)	0.0101 (9)	0.0227 (15)	0.0045 (8)	−0.0028 (11)	−0.0053 (11)
C2	0.0025 (11)	0.0164 (13)	0.0213 (11)	0.0031 (10)	−0.0014 (10)	−0.0057 (11)
C3	0.0102 (10)	0.0131 (13)	0.0249 (15)	0.0047 (9)	−0.0060 (10)	−0.0064 (11)
C4	0.0063 (9)	0.0131 (14)	0.0203 (16)	0.0024 (9)	−0.0003 (10)	0.0008 (10)
C5	0.0054 (11)	0.0101 (12)	0.0188 (13)	0.0024 (9)	0.0024 (10)	−0.0003 (8)
C6	0.0055 (10)	0.0106 (13)	0.0170 (13)	0.0016 (9)	−0.0002 (10)	0.0003 (8)
C7	0.0066 (11)	0.0168 (12)	0.0068 (13)	−0.0014 (10)	−0.0004 (10)	−0.0002 (10)
C8	0.0110 (13)	0.0190 (14)	0.0167 (15)	0.0005 (11)	−0.0013 (11)	−0.0035 (11)
C9	0.0068 (12)	0.0344 (17)	0.0160 (15)	0.0012 (12)	0.0035 (11)	−0.0031 (13)
C10	0.0102 (11)	0.0330 (17)	0.0204 (16)	−0.0083 (12)	0.0036 (12)	−0.0015 (12)
C11	0.0194 (14)	0.0174 (14)	0.0221 (18)	−0.0047 (10)	0.0044 (14)	−0.0019 (11)
C12	0.0075 (11)	0.0183 (13)	0.0165 (14)	0.0010 (9)	0.0039 (11)	−0.0007 (11)
C13	0.0109 (13)	0.0122 (14)	0.0169 (15)	−0.0022 (10)	−0.0018 (12)	−0.0015 (10)
C14	0.0197 (13)	0.0147 (14)	0.0214 (16)	0.0042 (11)	−0.0005 (12)	−0.0009 (11)
C15	0.0307 (15)	0.0172 (15)	0.0216 (16)	0.0063 (12)	−0.0041 (14)	0.0053 (12)
C16	0.0328 (16)	0.0238 (17)	0.0168 (15)	0.0005 (13)	−0.0025 (14)	0.0069 (12)
C17	0.0222 (14)	0.0193 (15)	0.0212 (16)	−0.0007 (12)	0.0021 (13)	−0.0026 (12)
C18	0.0130 (12)	0.0158 (14)	0.0179 (15)	0.0036 (10)	−0.0014 (11)	0.0000 (11)
C19	0.0029 (11)	0.0133 (13)	0.0150 (14)	−0.0030 (9)	−0.0007 (10)	0.0032 (10)
C20	0.0062 (11)	0.0155 (14)	0.0195 (15)	−0.0023 (9)	0.0007 (11)	0.0004 (11)
C21	0.0153 (13)	0.0180 (14)	0.0195 (15)	−0.0073 (11)	−0.0003 (12)	−0.0015 (11)
C22	0.0105 (13)	0.0271 (16)	0.0203 (16)	−0.0091 (11)	−0.0057 (11)	0.0040 (12)
C23	0.0042 (11)	0.0205 (15)	0.0277 (18)	−0.0022 (10)	−0.0043 (11)	0.0078 (12)
C24	0.0075 (11)	0.0140 (13)	0.0213 (15)	−0.0006 (10)	0.0011 (11)	0.0016 (11)
N1	0.0099 (11)	0.0202 (14)	0.0203 (14)	−0.0005 (9)	−0.0044 (11)	0.0018 (9)
C25	0.0109 (14)	0.0270 (16)	0.035 (2)	0.0028 (12)	−0.0101 (14)	0.0021 (13)
C26	0.0109 (15)	0.055 (2)	0.049 (3)	0.0007 (14)	−0.0023 (16)	0.0094 (18)
C27	0.0106 (12)	0.0254 (16)	0.0145 (15)	−0.0006 (11)	−0.0020 (11)	−0.0018 (11)
C28	0.0275 (16)	0.0240 (17)	0.0239 (18)	−0.0002 (12)	0.0005 (15)	−0.0023 (12)
C29	0.0290 (17)	0.0224 (17)	0.0129 (16)	0.0028 (12)	−0.0032 (14)	0.0020 (10)
C30	0.034 (2)	0.035 (2)	0.0235 (19)	0.0127 (15)	0.0101 (16)	0.0052 (13)
C31	0.0185 (13)	0.0220 (16)	0.0210 (16)	0.0006 (11)	−0.0055 (13)	0.0064 (12)
C32	0.040 (2)	0.0233 (18)	0.039 (2)	−0.0032 (14)	−0.0147 (17)	0.0053 (15)

### Geometric parameters (Å, °)

**Table:**

Mn1—C3	1.789 (3)	C17—H17	0.9500
Mn1—C2	1.797 (3)	C18—H18	0.9500
Mn1—C1	1.807 (3)	C19—C24	1.398 (4)
Mn1—O8	2.014 (2)	C19—C20	1.406 (4)
Mn1—O9	2.0184 (18)	C20—C21	1.392 (4)
Mn1—O7	2.0276 (18)	C20—H20	0.9500
Mn1—Mn2	2.8765 (5)	C21—C22	1.383 (4)
Mn2—C6	1.795 (3)	C21—H21	0.9500
Mn2—C4	1.796 (3)	C22—C23	1.397 (5)
Mn2—C5	1.812 (3)	C22—H22	0.9500
Mn2—O8	2.017 (2)	C23—C24	1.384 (4)
Mn2—O9	2.0284 (19)	C23—H23	0.9500
Mn2—O7	2.0444 (17)	C24—H24	0.9500
O1—C1	1.138 (4)	N1—C31	1.509 (4)
O2—C2	1.149 (4)	N1—C29	1.514 (4)
O3—C3	1.149 (3)	N1—C27	1.516 (4)
O4—C4	1.151 (4)	N1—C25	1.526 (4)
O5—C5	1.142 (3)	C25—C26	1.510 (6)
O6—C6	1.150 (3)	C25—H25A	0.9900
O7—C7	1.342 (3)	C25—H25B	0.9900
O8—C13	1.340 (4)	C26—H26A	0.9800
O9—C19	1.348 (3)	C26—H26B	0.9800
C7—C12	1.395 (4)	C26—H26C	0.9800
C7—C8	1.399 (4)	C27—C28	1.509 (4)
C8—C9	1.394 (4)	C27—H27A	0.9900
C8—H8	0.9500	C27—H27B	0.9900
C9—C10	1.365 (5)	C28—H28A	0.9800
C9—H9	0.9500	C28—H28B	0.9800
C10—C11	1.393 (4)	C28—H28C	0.9800
C10—H10	0.9500	C29—C30	1.509 (5)
C11—C12	1.393 (4)	C29—H29A	0.9900
C11—H11	0.9500	C29—H29B	0.9900
C12—H12	0.9500	C30—H30A	0.9800
C13—C14	1.404 (4)	C30—H30B	0.9800
C13—C18	1.403 (4)	C30—H30C	0.9800
C14—C15	1.382 (4)	C31—C32	1.517 (5)
C14—H14	0.9500	C31—H31A	0.9900
C15—C16	1.384 (5)	C31—H31B	0.9900
C15—H15	0.9500	C32—H32A	0.9800
C16—C17	1.388 (5)	C32—H32B	0.9800
C16—H16	0.9500	C32—H32C	0.9800
C17—C18	1.389 (4)		
			
C3—Mn1—C2	87.67 (13)	C16—C15—H15	119.4
C3—Mn1—C1	88.86 (12)	C15—C16—C17	118.7 (3)
C2—Mn1—C1	91.51 (13)	C15—C16—H16	120.7
C3—Mn1—O8	98.90 (12)	C17—C16—H16	120.7
C2—Mn1—O8	170.52 (11)	C18—C17—C16	120.6 (3)
C1—Mn1—O8	95.42 (11)	C18—C17—H17	119.7
C3—Mn1—O9	173.68 (11)	C16—C17—H17	119.7
C2—Mn1—O9	97.52 (10)	C17—C18—C13	121.3 (3)
C1—Mn1—O9	94.59 (10)	C17—C18—H18	119.3
O8—Mn1—O9	75.52 (8)	C13—C18—H18	119.3
C3—Mn1—O7	101.23 (10)	O9—C19—C24	121.3 (2)
C2—Mn1—O7	96.34 (11)	O9—C19—C20	120.9 (2)
C1—Mn1—O7	167.43 (9)	C24—C19—C20	117.8 (2)
O8—Mn1—O7	75.76 (8)	C21—C20—C19	120.7 (2)
O9—Mn1—O7	74.70 (7)	C21—C20—H20	119.7
C3—Mn1—Mn2	128.92 (9)	C19—C20—H20	119.7
C2—Mn1—Mn2	126.05 (9)	C22—C21—C20	121.3 (3)
C1—Mn1—Mn2	122.23 (8)	C22—C21—H21	119.4
O8—Mn1—Mn2	44.50 (6)	C20—C21—H21	119.4
O9—Mn1—Mn2	44.84 (5)	C21—C22—C23	118.1 (3)
O7—Mn1—Mn2	45.30 (5)	C21—C22—H22	121.0
C6—Mn2—C4	87.53 (12)	C23—C22—H22	121.0
C6—Mn2—C5	92.48 (12)	C24—C23—C22	121.4 (2)
C4—Mn2—C5	90.49 (12)	C24—C23—H23	119.3
C6—Mn2—O8	96.99 (10)	C22—C23—H23	119.3
C4—Mn2—O8	98.50 (10)	C23—C24—C19	120.8 (3)
C5—Mn2—O8	167.19 (10)	C23—C24—H24	119.6
C6—Mn2—O9	170.16 (9)	C19—C24—H24	119.6
C4—Mn2—O9	99.48 (10)	C31—N1—C29	111.4 (2)
C5—Mn2—O9	94.35 (11)	C31—N1—C27	106.2 (2)
O8—Mn2—O9	75.24 (8)	C29—N1—C27	111.5 (2)
C6—Mn2—O7	98.26 (10)	C31—N1—C25	110.7 (2)
C4—Mn2—O7	171.95 (10)	C29—N1—C25	106.2 (2)
C5—Mn2—O7	94.83 (10)	C27—N1—C25	110.9 (2)
O8—Mn2—O7	75.33 (7)	C26—C25—N1	115.4 (3)
O9—Mn2—O7	74.12 (7)	C26—C25—H25A	108.4
C6—Mn2—Mn1	125.60 (8)	N1—C25—H25A	108.4
C4—Mn2—Mn1	127.13 (8)	C26—C25—H25B	108.4
C5—Mn2—Mn1	122.77 (9)	N1—C25—H25B	108.4
O8—Mn2—Mn1	44.42 (6)	H25A—C25—H25B	107.5
O9—Mn2—Mn1	44.56 (5)	C25—C26—H26A	109.5
O7—Mn2—Mn1	44.82 (5)	C25—C26—H26B	109.5
C7—O7—Mn1	133.11 (15)	H26A—C26—H26B	109.5
C7—O7—Mn2	132.96 (16)	C25—C26—H26C	109.5
Mn1—O7—Mn2	89.89 (7)	H26A—C26—H26C	109.5
C13—O8—Mn1	133.21 (17)	H26B—C26—H26C	109.5
C13—O8—Mn2	135.63 (17)	C28—C27—N1	115.2 (2)
Mn1—O8—Mn2	91.07 (9)	C28—C27—H27A	108.5
C19—O9—Mn1	133.46 (16)	N1—C27—H27A	108.5
C19—O9—Mn2	135.87 (16)	C28—C27—H27B	108.5
Mn1—O9—Mn2	90.60 (8)	N1—C27—H27B	108.5
O1—C1—Mn1	178.8 (3)	H27A—C27—H27B	107.5
O2—C2—Mn1	179.0 (2)	C27—C28—H28A	109.5
O3—C3—Mn1	176.3 (2)	C27—C28—H28B	109.5
O4—C4—Mn2	176.2 (3)	H28A—C28—H28B	109.5
O5—C5—Mn2	177.1 (2)	C27—C28—H28C	109.5
O6—C6—Mn2	176.8 (2)	H28A—C28—H28C	109.5
O7—C7—C12	120.6 (2)	H28B—C28—H28C	109.5
O7—C7—C8	121.1 (2)	C30—C29—N1	114.8 (3)
C12—C7—C8	118.3 (2)	C30—C29—H29A	108.6
C9—C8—C7	120.4 (3)	N1—C29—H29A	108.6
C9—C8—H8	119.8	C30—C29—H29B	108.6
C7—C8—H8	119.8	N1—C29—H29B	108.6
C10—C9—C8	121.2 (3)	H29A—C29—H29B	107.5
C10—C9—H9	119.4	C29—C30—H30A	109.5
C8—C9—H9	119.4	C29—C30—H30B	109.5
C9—C10—C11	119.0 (3)	H30A—C30—H30B	109.5
C9—C10—H10	120.5	C29—C30—H30C	109.5
C11—C10—H10	120.5	H30A—C30—H30C	109.5
C12—C11—C10	120.7 (3)	H30B—C30—H30C	109.5
C12—C11—H11	119.7	N1—C31—C32	115.9 (3)
C10—C11—H11	119.7	N1—C31—H31A	108.3
C11—C12—C7	120.4 (2)	C32—C31—H31A	108.3
C11—C12—H12	119.8	N1—C31—H31B	108.3
C7—C12—H12	119.8	C32—C31—H31B	108.3
O8—C13—C14	121.0 (3)	H31A—C31—H31B	107.4
O8—C13—C18	121.8 (2)	C31—C32—H32A	109.5
C14—C13—C18	117.2 (3)	C31—C32—H32B	109.5
C15—C14—C13	121.1 (3)	H32A—C32—H32B	109.5
C15—C14—H14	119.5	C31—C32—H32C	109.5
C13—C14—H14	119.5	H32A—C32—H32C	109.5
C14—C15—C16	121.2 (3)	H32B—C32—H32C	109.5
C14—C15—H15	119.4		
			
C3—Mn1—Mn2—C6	1.44 (17)	C5—Mn2—O8—Mn1	1.9 (5)
C2—Mn1—Mn2—C6	−120.10 (14)	O9—Mn2—O8—Mn1	38.38 (7)
C1—Mn1—Mn2—C6	120.06 (16)	O7—Mn2—O8—Mn1	−38.70 (7)
O8—Mn1—Mn2—C6	58.90 (13)	C2—Mn1—O9—C19	47.5 (3)
O9—Mn1—Mn2—C6	−179.94 (14)	C1—Mn1—O9—C19	−44.6 (3)
O7—Mn1—Mn2—C6	−61.99 (13)	O8—Mn1—O9—C19	−139.1 (2)
C3—Mn1—Mn2—C4	−116.94 (19)	O7—Mn1—O9—C19	142.1 (2)
C2—Mn1—Mn2—C4	121.51 (16)	Mn2—Mn1—O9—C19	−177.3 (3)
C1—Mn1—Mn2—C4	1.68 (18)	C2—Mn1—O9—Mn2	−135.16 (10)
O8—Mn1—Mn2—C4	−59.49 (15)	C1—Mn1—O9—Mn2	132.70 (11)
O9—Mn1—Mn2—C4	61.67 (15)	O8—Mn1—O9—Mn2	38.28 (7)
O7—Mn1—Mn2—C4	179.62 (16)	O7—Mn1—O9—Mn2	−40.61 (8)
C3—Mn1—Mn2—C5	123.05 (16)	C4—Mn2—O9—C19	42.6 (3)
C2—Mn1—Mn2—C5	1.50 (14)	C5—Mn2—O9—C19	−48.6 (3)
C1—Mn1—Mn2—C5	−118.33 (15)	O8—Mn2—O9—C19	138.9 (3)
O8—Mn1—Mn2—C5	−179.49 (12)	O7—Mn2—O9—C19	−142.4 (3)
O9—Mn1—Mn2—C5	−58.33 (13)	Mn1—Mn2—O9—C19	177.2 (3)
O7—Mn1—Mn2—C5	59.62 (13)	C4—Mn2—O9—Mn1	−134.64 (11)
C3—Mn1—Mn2—O8	−57.46 (15)	C5—Mn2—O9—Mn1	134.13 (9)
C2—Mn1—Mn2—O8	−179.00 (12)	O8—Mn2—O9—Mn1	−38.27 (7)
C1—Mn1—Mn2—O8	61.16 (14)	O7—Mn2—O9—Mn1	40.34 (7)
O9—Mn1—Mn2—O8	121.16 (11)	Mn1—O7—C7—C12	−169.8 (2)
O7—Mn1—Mn2—O8	−120.89 (11)	Mn2—O7—C7—C12	−19.6 (4)
C3—Mn1—Mn2—O9	−178.62 (16)	Mn1—O7—C7—C8	11.9 (4)
C2—Mn1—Mn2—O9	59.84 (12)	Mn2—O7—C7—C8	162.1 (2)
C1—Mn1—Mn2—O9	−60.00 (14)	O7—C7—C8—C9	−179.5 (3)
O8—Mn1—Mn2—O9	−121.16 (11)	C12—C7—C8—C9	2.2 (4)
O7—Mn1—Mn2—O9	117.95 (12)	C7—C8—C9—C10	0.2 (5)
C3—Mn1—Mn2—O7	63.43 (15)	C8—C9—C10—C11	−2.1 (5)
C2—Mn1—Mn2—O7	−58.11 (13)	C9—C10—C11—C12	1.7 (5)
C1—Mn1—Mn2—O7	−177.95 (15)	C10—C11—C12—C7	0.7 (5)
O8—Mn1—Mn2—O7	120.89 (11)	O7—C7—C12—C11	179.0 (3)
O9—Mn1—Mn2—O7	−117.95 (12)	C8—C7—C12—C11	−2.6 (4)
C3—Mn1—O7—C7	23.9 (3)	Mn1—O8—C13—C14	7.0 (4)
C2—Mn1—O7—C7	−65.0 (2)	Mn2—O8—C13—C14	−177.19 (19)
C1—Mn1—O7—C7	166.7 (5)	Mn1—O8—C13—C18	−173.50 (19)
O8—Mn1—O7—C7	120.3 (2)	Mn2—O8—C13—C18	2.3 (4)
O9—Mn1—O7—C7	−161.1 (2)	O8—C13—C14—C15	179.6 (3)
Mn2—Mn1—O7—C7	158.7 (3)	C18—C13—C14—C15	0.1 (4)
C3—Mn1—O7—Mn2	−134.81 (11)	C13—C14—C15—C16	−0.3 (5)
C2—Mn1—O7—Mn2	136.31 (10)	C14—C15—C16—C17	0.0 (5)
C1—Mn1—O7—Mn2	8.0 (6)	C15—C16—C17—C18	0.4 (5)
O8—Mn1—O7—Mn2	−38.36 (8)	C16—C17—C18—C13	−0.6 (5)
O9—Mn1—O7—Mn2	40.22 (8)	O8—C13—C18—C17	−179.2 (3)
C6—Mn2—O7—C7	−25.2 (3)	C14—C13—C18—C17	0.3 (4)
C5—Mn2—O7—C7	68.0 (2)	Mn1—O9—C19—C24	170.61 (19)
O8—Mn2—O7—C7	−120.3 (2)	Mn2—O9—C19—C24	−5.6 (4)
O9—Mn2—O7—C7	161.2 (2)	Mn1—O9—C19—C20	−10.7 (4)
Mn1—Mn2—O7—C7	−158.7 (3)	Mn2—O9—C19—C20	173.16 (19)
C6—Mn2—O7—Mn1	133.50 (10)	O9—C19—C20—C21	−178.5 (2)
C5—Mn2—O7—Mn1	−133.28 (10)	C24—C19—C20—C21	0.3 (4)
O8—Mn2—O7—Mn1	38.38 (8)	C19—C20—C21—C22	−0.4 (4)
O9—Mn2—O7—Mn1	−40.12 (8)	C20—C21—C22—C23	−0.1 (4)
C3—Mn1—O8—C13	−44.6 (2)	C21—C22—C23—C24	0.6 (4)
C1—Mn1—O8—C13	45.1 (2)	C22—C23—C24—C19	−0.7 (4)
O9—Mn1—O8—C13	138.5 (2)	O9—C19—C24—C23	179.0 (2)
O7—Mn1—O8—C13	−144.0 (2)	C20—C19—C24—C23	0.2 (4)
Mn2—Mn1—O8—C13	177.0 (3)	C31—N1—C25—C26	−61.1 (4)
C3—Mn1—O8—Mn2	138.41 (10)	C29—N1—C25—C26	177.9 (3)
C1—Mn1—O8—Mn2	−131.90 (10)	C27—N1—C25—C26	56.5 (4)
O9—Mn1—O8—Mn2	−38.55 (7)	C31—N1—C27—C28	−179.5 (2)
O7—Mn1—O8—Mn2	39.00 (7)	C29—N1—C27—C28	−58.0 (3)
C6—Mn2—O8—C13	47.6 (3)	C25—N1—C27—C28	60.2 (3)
C4—Mn2—O8—C13	−40.9 (3)	C31—N1—C29—C30	60.3 (3)
C5—Mn2—O8—C13	−175.0 (4)	C27—N1—C29—C30	−58.1 (3)
O9—Mn2—O8—C13	−138.5 (2)	C25—N1—C29—C30	−179.0 (3)
O7—Mn2—O8—C13	144.4 (3)	C29—N1—C31—C32	56.2 (3)
Mn1—Mn2—O8—C13	−176.9 (3)	C27—N1—C31—C32	177.8 (3)
C6—Mn2—O8—Mn1	−135.45 (9)	C25—N1—C31—C32	−61.7 (4)
C4—Mn2—O8—Mn1	136.02 (11)		
